# Differences in Ventilatory Threshold for Exercise Prescription in Outpatient Diabetic and Sarcopenic Obese Subjects

**DOI:** 10.1155/2016/6739150

**Published:** 2016-01-14

**Authors:** Gian Pietro Emerenziani, Maria Chiara Gallotta, Silvia Migliaccio, Emanuela A. Greco, Chiara Marocco, Luca di Lazzaro, Rachele Fornari, Andrea Lenzi, Carlo Baldari, Laura Guidetti

**Affiliations:** ^1^Department of Movement, Human and Health Sciences, University of Rome “Foro Italico”, Piazza Lauro de Bosis 6, 00135 Rome, Italy; ^2^Department of Experimental Medicine, Sapienza University of Rome, Rome, Italy; ^3^LiSa Laboratory, Policlinico of Catania, Universitario of Catania, Catania, Italy

## Abstract

Aim of the study was to examine cardiorespiratory parameters at individual ventilatory threshold (IVT) and peak exercise capacity (${\dot{V}O}_{2peak}$) in outpatient diabetic and sarcopenic obese subjects. Seventeen obese subjects (BMI: 36.6 ± 4.1 kg·m^−1^) and sixteen SO subjects (BMI: 37.0 ± 7.3 kg·m^−1^) were compared with sixteen T2DM subjects (BMI: 37.7 ± 5.6 kg·m^−1^). All groups performed an incremental exercise test on a treadmill according to their physical ability. ${\dot{V}O}_{2peak}$, %HR_max_, and maximal metabolic equivalent (MET_max_) were evaluated at maximal effort. Moreover, ${\dot{V}O}_{2ivt}$, %$\dot{V}O_{2peak}$, %HR_max_, %HRR, ΔHR, and METivt were assessed at IVT. No significant differences were found in any physiological parameters at maximal effort (${\dot{V}O}_{2peak}$, %HR_max_, and MET_max_) in all groups. On the contrary, ${\dot{V}O}_{2ivt}$, %${\dot{V}O}_{2peak}$, %HR_max_, %HRR, ΔHR, and MET_ivt_ were significantly lower in T2DM subjects as compared to OB and SO subjects at IVT (*p* < 0.05). Our results show that while at maximal effort there are no differences among groups, at IVT the physiological parameters are lower in T2DM subjects than in OB and SO subjects. Therefore, due to the differences observed in the groups, we suggest usng the IVT as a useful parameter to prescribe aerobic exercise in obese with sarcopenia or diabetes mellitus conditions.

## 1. Introduction

Obesity is one of the greatest public health challenges of the 21st century. Between 1980 and 2008, the worldwide prevalence of obesity almost doubled [1]. Behavioral risk factors including physical inactivity and unhealthy diet are estimated to be responsible for about 80% of coronary heart disease and cerebrovascular disease [1]. Obesity drastically increases the individual risk of developing chronic metabolic diseases, such as cardiovascular disease, cancer, and diabetes mellitus. In particular, type 2 diabetes mellitus (T2DM) is a worldwide pathological condition associated with obesity and sedentary lifestyle [2]. Interestingly, adipose tissue excess and physical inactivity have led to the development of a pathological condition defined as “sarcopenic obesity.” Hence, sarcopenic obesity prevalence ranges from 2.75% to over 20%, depending on the criteria used for the diagnosis and methods of body composition assessment [3]. Sarcopenia has been described as age-related loss of muscle mass that results in decreased strength, aerobic power, and functional capacity [4, 5]. Thus, sarcopenic obesity condition is commonly observed in older people [6] but, more recently, it can also be observed in younger adults, likely linked to physical inactivity and sedentary behavior [6, 7].

Moreover, recent data have demonstrated that obese T2DM patients have an impaired muscle strength which could increase adverse events in these subjects [8].

Physical activity (PA), along with correct nutritional intervention, is considered crucial for the prevention and management of both sarcopenic obesity and diabetes. Indeed, it is well known that regular PA is an essential component in primary and secondary prevention for most of the NCDs since it provides several health benefits [9, 10]. PA treatment goals in obese patients with sarcopenia or diabetes include achievement of optimal body composition, aerobic and functional capacity. It is well known that exercise is more efficient when is structured and performed after a precise prescription and with serious coaching and follow-up. However, few issues need to be further evaluated and characterized when PA is prescribed to obese individuals. For instance, the role of exercise intensity in physical training adherence might play a fundamental role [11, 12]. Individualizing the exercise intensity after an exercise test in order to perform moderate intensity it has been reported to provide positive results [9].

Indeed, exercise intensity should be set to reach positive physiological effects, a decreased risk of injury, and increased adherence.

PA can elicit different responses in obese individuals when prescribed in absolute terms, such as gait speed and power [13], since no individual adjustment is usually made for each subject's exercise capacity. Moreover, the use of relative terms such as %$\dot{V}O_{2max}$ and %HR_max_ has been substantially criticized [9, 14, 15] since it seems that the relative parameters alone, without considering the individual ventilatory threshold (IVT), are not sufficient and adequate to individualize exercise intensity [9]. In particular, IVT is the point after which ventilation begins to increase disproportionately relative to oxygen uptake so that it was called “point of optimal ventilatory efficiency” [16]. It is considered a useful submaximal breakpoint for optimal moderate exercise intensity prescription in T2DM patients and obese subjects [17, 18]. The work load corresponding to these physiological events is sometimes termed “ventilatory threshold 1” or “aerobic threshold” [15]; we will use the term “individual ventilatory threshold” (IVT) for the rest of the text. It is possible that different pathological conditions could influence physiological responses to exercise. For instance, Metz et al. [19] showed that T2DM patients have alterations in muscle substrate utilization. For T2DM patients, predicting the IVT using only relative terms could not lead the specialist physical activity teacher to select the exercise intensity for optimal moderate intensity training. Seeing that the exercise intensity to perform moderate intensity training could be modified by a different pathological condition in obese subjects, it is very important to individualize the intensity of exercise after an individualized exercise test. This approach may permit individualizing the target intensity training according to the different subjects' pathological conditions. Moreover, Dishman and Buckworth [20] showed that activity-promotion efforts were more effective when the intensity of PA was low rather than high. Previous studies [21, 22] showed that subjects would be more inclined to choose an exercise intensity around their IVT.

No data are available to date regarding exercise capacity in adult affected by sarcopenic obesity. As result, we might hypothesize that differences could exist in responses during exercise among different obesity conditions at the same relative exercise intensity.

Therefore, aim of our study was to evaluate whether differences could exist on physiological parameters at IVT and at maximal effort in obese with sarcopenia or diabetes mellitus subjects.

## 2. Methods

### 2.1. Participants

Sixteen female obese subjects (mean ± SD: age: 58.8 ± 7.8 yrs), sixteen female obese subjects with type 2 diabetes (age: 60.0 ± 10.0 yrs), and sixteen female sarcopenic obese individuals (age: 60.9 ± 13.0 yrs) were selected in the Department of Experimental Medicine, Section of Medical Pathophysiology, Food Science and Endocrinology, the Policlinico Umberto I, Sapienza University of Rome between January 2013 and June 2013. Patients were divided into the groups based on the diagnosis of type 2 diabetes mellitus or sarcopenia (as described in the following paragraph). Participants provided written informed consent and protocol was approved by the Local Ethical Committee of Policlinico Umberto I, Sapienza University of Rome. All subjects were sedentary and they had not been previously engaged in regular physical exercise. Subjects underwent clinical examination to rule out any contraindications to PA (such as neuropathy, autonomic dysfunction, cardiovascular diseases, and blood pressure ≥140/90 mmHg).

### 2.2. Clinical Evaluation

All subjects were evaluated in the morning. Participants' height was measured using a stadiometer to the nearest 0.1 cm. Body weight (BW) measurements were taken on a calibrated digital scale (InBody 720, Biospace Inc., Cerritos, CA, USA) [23] with underwear clothing. Body mass index (BMI) was calculated as BW divided by squared height (kg/m^2^). Diagnosis of type 2 diabetes mellitus (T2DM) was established according to the World Health Organization criteria [24] and T2DM patients were on oral pharmacological treatments. Obesity was diagnosed as fat mass equal to or above 35% in women and 25% in men. Sarcopenia was diagnosed according to the definition by the European Working Group on Sarcopenia in Older People (EWGSOP), considering the reduction of muscle mass, the impairment of muscle strength, and physical performance [6]. FFM was considered low if a patient's fat-free mass (FFM) was <90% of their ideal FFM (iFFM). Ideal FFM was calculated in kg as the sum of 75% of ideal body weight and 25% of overweight, as expressed by a BMI > 24.9 kg/m^2^ [5, 10]. Body composition was evaluated by DEXA (Hologic, Bedford, United States) and by Body Impedance Assessment (BIA 101 Impedance Analyzer, Akern, Florence, Italy) [25]. Dietary counselling and psychological counselling were performed by a dietitian and a psychologist, respectively. A low-calorie diet was set at approximately 400Kcal less than total daily energy expenditure in all patients. Total daily energy expenditure was set according to the following equation: resting metabolic rate + PA level. Resting metabolic rate was estimated by the Harris Benedict equation [26] while PA level was estimated by the International Physical Activity Questionnaire (IPAQ) [27].

Pulmonary function tests and a resting electrocardiogram (ECG) recording were performed as initial screening in all patients. Prior to the test session, participants took part in a familiarization session to become accustomed to tests. A graded incremental exercise test was performed as described in the following paragraph.

### 2.3. Maximal Effort and Individual Ventilatory Threshold

$\dot{V}O_{2peak}$ was assessed in all participants by means of a continuous, maximal graded exercise test on a treadmill according to individual abilities [18]. In particular, subjects performed an incremental protocol [18] on a treadmill (Pro treadmill, Woodway, Waukesha, WI, USA). During exercise testing, subjects were asked to report the degree of exertion every two minutes, based on the RPE scale [28]. Treadmill protocol started at 3 km/h and then speed increased by 1 km/h every two minutes until 5 km/h was reached. Then, slope was increased by 3% every two minutes until subjects reached a value of 10 on RPE scale [28]. Heart rate (beats·min^−1^) was continuously recorded before and throughout the trial using a HR monitor (RS 400, Polar Electro, Kempele, Finland). $\dot{V}O_{2}$ and pulmonary ventilation ($\dot{V}E$) were measured by a semiportable gas analysis system (FitMate pro, Cosmed, Rome, Italy) [29]. Prior to each test, the Fitmate pro underwent an automatic gas calibration cycle and the turbine flow meter was periodically calibrated using a 3 L syringe according to the manufacturer's recommendations. During the test, the highest $\dot{V}O_{2}$ attained was chosen as the peak oxygen uptake ($\dot{V}O_{2peak}$). The individual ventilatory threshold (IVT) was determined offline for each subject by plotting the ventilatory equivalent ($\dot{V}E/\dot{V}O_{2}$) as a function of $\dot{V}O_{2}$ to identify the point during exercise where this curve reached its lowest value [9, 16, 30]. The level of $\dot{V}O_{2}$ at which we observed the lowest value of $\dot{V}E/\dot{V}O_{2}$, in the individual plot, was the IVT [31]. The exercise intensity (speed and slope) corresponding to IVT was reached by all the subjects. At IVT and at maximal effort, heart rate (HR) and metabolic equivalent (MET) were measured. In addition, ΔHR (HR at IVT − HR at rest), %HR_max_ [(HR at IVT)/(220 − age)], and %HRR [(HR at IVT − HR at rest)/(220 – age − HR at rest)] were also calculated.

### 2.4. Statistical Analysis

Based on a previous clinical trial [18], sample size estimation of 48 subjects achieved 81% power to detect differences among the means versus the alternative of equal means using an *F* test with a 0.05 significance level. Normal distribution of data was tested by calculating the skewness and kurtosis and by employing the Kolmogorov-Smirnov test. Homogeneity of variance was confirmed by Levine's test. The results obtained in the study are expressed as means ± standard deviation (SD). To verify that groups did not differ among each other regarding age, weight, height, BMI, and %FM, one-way ANOVA was performed. Moreover, one-way ANOVA was used to assess differences among groups at maximal effort and at IVT. Differences between groups were evaluated with a multiple unpaired *t*-test with Bonferroni correction. All statistical analyses were performed with the SPSS statistical package (Version 20.0 for Windows; SPSS Inc., Chicago, IL, USA). All tests were two-tailed, with *α* = 0.05 being taken as significant.

## 3. Results

All subjects were evaluated before being enrolled in the study. Anthropometric measurements were taken as described in the materials and methods section. In particular, age and BW were similar in different groups as depicted in Table 1.

Further biochemical evaluation showed that glycated hemoglobin was 49.9 ± 8.3 mmol/mol in T2DM group, while no alterations in glycemic values were found in subjects of the other 2 groups. As previously mentioned, treadmill protocol started at 3 km/h and then speed increased by 1 km/h every 2 minutes until 5 km/h was reached, while slope was increased by 3% every minute until subjects reached a value of 10 on RPE scale. The results regarding absolute and relative exercise intensities at maximal effort in obese, sarcopenic obese, and T2DM subjects are shown in Tables 2 and 3.

The subjects $\dot{V}O_{2peak}$ ranging from 18.1 mL min^−1^ kg^−1^ in OB, 16.9 mL min^−1^ kg^−1^ in SO, and 16.7 mL min^−1^ kg^−1^ in T2DM groups demonstrate that the subjects were very unfit. In particular, no significant differences were found among the three different groups for any studied variable at maximal effort as shown in Table 2.

All subjects reached IVT and no significant adverse events were observed during the testing session in all groups (Table 2).

Interestingly, all the variables at IVT demonstrated significant differences in T2DM subjects group as compared to the other two groups of obese individuals, as depicted in Table 3. In detail, at IVT all the studied variables were significantly lower in T2DM groups than in OB and OS groups indicating that T2DM subjects had significantly lower exercise capacity at IVT. At IVT no significant differences were found between OB and OS group.

Data regarding peak exercise capacity of these subjects showed that they were all very untrained and unfit.

## 4. Discussion

The primary finding of our study was that all the physiological parameters considered for PA at IVT were lower in T2DM group as compared to OB and SO groups, even if at maximal effort no differences were observed among groups.

Thus, the results obtained in our study demonstrate the importance of the use of IVT to prescribe aerobic exercise training in different obesity conditions.

T2DM subjects were obese and they had not practiced any organized PA prior to the enrollment of this study. Moreover, subjects' fitness parameters, such as $\dot{V}O_{2peak}$, showed that they were unfit as previously described for obese population [32]. According to these observations, the use of a constant moderate intensity exercise as training method appears probably more appropriate. In fact, the American College of Sports Medicine (ACSM) PA guidelines for T2DM subjects recommend performing low-to-moderate intensity PA (at 40–70%$\;\;\dot{V}O_{2max}$) to achieve cardiorespiratory and metabolic improvement. More importantly, lower intensity activity leads to a more comfortable level of exertion and, likely, enhances the likelihood of adherence. At the same time it might reduce the risk of musculoskeletal injury and feet-ankle trauma, particularly when weight-bearing activity is recommended.

At present, ACSM suggests using three variables to monitor exercise intensity: $\dot{V}O_{2max}$, HR, and rate of perceived exertion (RPE). Moreover, during the last years, the gas exchange threshold was identified as valid tool to delineate the “training zone” for endurance training and for unhealthy subjects [15].

Indeed, peak exercise capacity of these subjects indicated that they were very untrained. In fact, $\dot{V}O_{2peak}$ was ranging from 11.9 to 21.5 mL min^−1^ kg^−1^ when considering all groups. Moreover during submaximal exercise test subjects did not reach their HR_max_ (220 − age) but they stopped around 75% of their maximal HR. Interestingly, our results demonstrate that when exercise intensity is prescribed according only to relative parameter based upon HR_max_ (such us %HR_max_ or %HRR), without adjustment for each individual's exercise capacity, exercise intensity could be overestimated. Our results, at peak exercise, were not different from those reported by Hulens et al. [32] in obese population confirming a lower physical fitness level in obese subjects. Low $\dot{V}O_{2peak}$ is in agreement with the literature regarding the cardiorespiratory fitness in obese subjects [32, 33]. Hulens et al. [32] showed that $\dot{V}O_{2peak}$ was 15.8 ± 3.8 mL min^−1^ kg^−1^ in obese women while in the study developed by Aiello et al. [33] the $\dot{V}O_{2peak}$ was around 15.8 mL min^−1^ kg^−1^. $\dot{V}O_{2peak}$ in T2DM subjects was previously described by our group [18] assessing that $\dot{V}O_{2peak}$ in T2DM subjects ranging from 15.9 to 18.6 mL min^−1^ kg^−1^. Our obese subjects, either sarcopenic or diabetic, had 16.9 ± 2.9 and 16.7 ± 4.8 mL min^−1^ kg^−1^, respectively, demonstrating that $\dot{V}O_{2peak}$ in SO and T2DM subjects does not differ from obese subjects. Moreover, ventilation and absolute $\dot{V}O_{2}$ were very low and no differences were present among groups, which could be explained by the influence of fat mass on respiratory system [32]. Even if the impact of obesity on cardiorespiratory fitness is not completely understood, fat mass might affect respiratory and exercise physiology during exercise. Respiratory muscle function and lung volumes at rest and during exercise could be potentially compromised as a result of obesity, influencing the breathlessness perception and the $\dot{V}O_{2peak}$ values. In fact, obese individuals show decreased respiratory system compliance, which results in a restrictive ventilatory defect. $\dot{V}O_{2peak}$ in T2DM patients is slightly lower than those previously reported [34], which could be explained by the younger age of the subjects recruited by Belli et al. [34] as compared to our study.

Regarding the data of $\dot{V}O_{2peak}$ in OS subjects, the results obtained in our study are the first ever described in such population. We might speculate that a decrease in muscle mass could have negative effects on $\dot{V}O_{2peak}$. However, there were no differences among the groups, which could be explained by a potential important negative effect of fat mass on $\dot{V}O_{2peak}$ in adult population [33]. T2DM group showed significantly lower results for all physiological parameters regarding exercise capacity at IVT, compared to OB and OS groups, suggesting that during exercise T2DM subjects reach the IVT earlier than OB and OS subjects. This result leads to a lower exercise capacity at IVT. The differences between T2DM group and the other two groups could be justified by the altered substrate utilization of T2DM subjects. In fact, it has been shown that T2DM patients have a greater boost in basal lactate levels than OB subjects [34]. Moreover, Metz et al. [19] showed that basal- and exercise-induced increases of lactate level in T2DM women were negatively correlated with 2 indexes of substrate utilization (crossover and maximal fat oxidation points). The crossover point of substrate utilization is defined as the power output at which energy from CHO-derived fuels predominates over energy from lipids, with further increases in power eliciting a relative increment in CHO utilization and a decrement in lipid oxidation. Differently, maximal fat oxidation point marks the exercise intensity at which the rate of fat oxidation is maximal. Metz et al. [19] hypothesized that an elevated lactate level could potentially act on substrate utilization by two mechanisms. First, it is the process of acting by promoting lactate utilization and, in the same way, carbohydrate oxidation. Consequently, T2DM subjects could have more pronounced carbohydrate utilization during exercise (lower crossover point). Secondly, lactate could inhibit lipid oxidation as already suggested in experimental animal models (low maximal fat oxidation point) [35].

The IVT marks the upper limit after which there is the first in blood lactate concentration that leads to slight rising in carbon dioxide partial pressure with a compensatory increased stimulus for ventilation [36]. The strong relationship between ventilatory threshold and substrate utilization could justify the lower physiological parameter values at IVT in T2DM group than in the other two groups.

These results highlight the importance to individualize the exercise intensity according to the individuals' physiological characteristics. In fact, T2DM showed a lower value of exercise intensity corresponding to IVT compared to OB and OS groups. Therefore, the specialist physical activity teacher could overestimate the exercise intensity for optimal moderate training if this intensity is evaluated according to relative terms $(\%{\dot{V}O}_{2max}$, %HR_max_, etc.)

In conclusion, even if there were no differences between T2DM and OB and OS groups at maximal effort, T2DM subjects showed a lower exercise capacity at IVT. The use of IVT should become more frequent in obese individuals, since training at this intensity improves aerobic capacity, cost effectiveness of treatment but might also increase adherence to physical activity in obese subjects. These novel results highlight the importance to prescribe exercise intensity, according to IVT to avoid the possibility of overestimating exercise intensity in obese subjects with different condition and, thus, we believe that they might be useful for professional figures who work to achieve and maintain fitness in obese population.

## Figures and Tables

**Table 1:** Physical characteristics of patients.

	OB	OS	T2DM
Age (yrs)	58.8 ± 7.8	59.6 ± 10.4	60.9 ± 13.0
Weight (kg)	91.4 ± 12.7	91.7 ± 23.0	99.1 ± 18.5
Height (cm)	157.8 ± 4.5	158.0 ± 5.5	161.9 ± 8.2
BMI (kg/m^2^)	36.6 ± 4.1	37.0 ± 7.3	37.7 ± 5.6
%FM (%)	43.5 ± 3.0	39.6 ± 5.5	41.0 ± 8.9
HR_rest_ (bpm)	78.5 ± 9.7	78.3 ± 15.1	76.3 ± 14.3

OB: obese subjects; OS: sarcopenic obese subjects; T2DM: type 2 diabetes mellitus; BMI: body mass index; HR_rest_: resting heart rate. No statistically significant difference (*p* < 0.05) was found between OB, OS, and T2DM subjects. Values are mean ± SD.

**Table 2:** Absolute and relative exercise intensities at maximal effort in obese, sarcopenic obese, and T2DM subjects.

	OB	OS	T2DM
Ventilation (L min^−1^)	50.0 ± 7.8	51.7 ± 7	48.5 ± 5.8
${\dot{V}O}_{\text{2peak}}$ (mL kg^−1^ min^−1^)	18.1 ± 3.2	16.9 ± 2.9	16.7 ± 4.8
${\dot{V}O}_{\text{2peak}}$ (mL min^−1^)	1711 ± 462	1517 ± 319	1706 ± 616
MET_peak_	5.2 ± 0.9	4.8 ± 0.8	4.8 ± 1.4
%HR_max⁡_	81.2 ± 9.6	75.3 ± 11.5	76.3 ± 9.3

Notes: OB: obese subjects; OS: sarcopenic obese subjects; T2DM: type 2 diabetes mellitus; ${\dot{V}O}_{\text{2peak}}$: peak oxygen uptake; MET: metabolic equivalent; %HR_max⁡_: percent of maximal heart rate. No statistically significant difference (*p* < 0.05) was found between OB, OS, and T2DM subjects.

**Table 3:** Absolute and relative exercise intensities at IVT in obese, sarcopenic obese, and T2DM subjects.

	OB	OS	T2DM
$\dot{V}O_{2ivt}$ (ml min^−1^ kg^−1^)	12.2 ± 2.5	11.6 ± 2.3	9.5 ± 2.6^*∗*^
%${\dot{V}O}_{\text{2peak}}$ (%)	67.6 ± 8.2	69.6 ± 8.5	58.5 ± 14.2^*∗*^
MET_ivt_	3.5 ± 0.7	3.3 ± 0.7	2.7 ± 0.8^*∗*^
%HR_max⁡_ (%)	69.1 ± 8.6	66.1 ± 10.2	58.0 ± 8.4^*∗*^
%HRR_ivt_ (%)	39.6 ± 16.4	33.4 ± 17.7	19.9 ± 9.9^*∗*^
ΔHR (bpm)	33.5 ± 14.2	27.6 ± 14.8	16.2 ± 7.8^*∗*^

OB: obese subjects; OS: sarcopenic obese subjects; T2DM: type 2 diabetes mellitus; $\dot{V}O_{2ivt}$: oxygen uptake; MET: metabolic equivalent. Data shown are intended as mean ± SD. ^*∗*^Statistically significant differences between T2DM subjects versus OB and OS groups (*p* < 0.05).
